# Serious Game Platform as a Possibility for Home-Based Telerehabilitation for Individuals With Cerebral Palsy During COVID-19 Quarantine – A Cross-Sectional Pilot Study

**DOI:** 10.3389/fpsyg.2021.622678

**Published:** 2021-02-02

**Authors:** Talita Dias da Silva, Paula Lumy da Silva, Elisa de Jesus Valenzuela, Eduardo Dati Dias, Amanda Orasmo Simcsik, Mariana Giovanelli de Carvalho, Anne Michelli Gomes Gonçalves Fontes, Camila Aparecida de Oliveira Alberissi, Luciano Vieira de Araújo, Murilo Vinícius da Costa Brandão, Helen Dawes, Carlos Bandeira de Mello Monteiro

**Affiliations:** ^1^Departamento de Medicina (Cardiologia), Escola Paulista de Medicina da Universidade Federal de São Paulo (UNIFESP), São Paulo, Brazil; ^2^Grupo de Pesquisa e Aplicações Tecnológicas em Reabilitação da Escola de Artes, Ciências e Humanidades da Universidade de São Paulo (PATER EACH USP), São Paulo, Brazil; ^3^Faculdade de Medicina, Universidade Cidade de São Paulo (UNICID), São Paulo, Brazil; ^4^Fundação Hermínio Ometto (FHO-UNIARARAS), São Paulo, Brazil; ^5^Departamento de Fisioterapia, Fonoaudiologia e Terapia Ocupacional, Faculdade de Medicina, Universidade de São Paulo (FOFITO – USP), São Paulo, Brazil; ^6^Institute of Nursing and Allied Health Research, Oxford Brookes University, Oxford, United Kingdom; ^7^Department of Clinical Neurology, University of Oxford, Oxford, United Kingdom

**Keywords:** cerebral palsy, motor rehabilitation, telerehabilitation, physical functional performance, serious game

## Abstract

**Introduction:**

There is a need to maintain rehabilitation activities and motivate movement and physical activity during quarantine in individuals with Cerebral Palsy (CP).

**Objective:**

This paper sets out to evaluate the feasibility and potential benefits of using computer serious game in a non-immersive virtual reality (VR) implemented and evaluated completely remotely in participants with CP for Home-Based Telerehabilitation during the quarantine period for COVID-19.

**Methods:**

Using a cross-sectional design, a total of 44 individuals participated in this study between March and June 2020, 22 of which had CP (14 males and 8 females, mean age = 19 years, ranging between 11 and 28 years) and 22 typically developing individuals, matched by age and sex to the individuals with CP. Participants practiced a coincident timing game^1^ and we measured movement performance and physical activity intensity using the rating of perceived exertion Borg scale.

**Results:**

All participants were able to engage with the VR therapy remotely, reported enjoying sessions, and improved performance in some practice moments. The most important result in this cross-sectional study was the significant increasing in rating of perceived exertion (through Borg scale) in both groups during practice and with CP presenting a higher rating of perceived exertion.

**Conclusion:**

Children with CP enjoyed participating, were able to perform at the same level as their peers on certain activities and increased both their performance and physical activity intensity when using the game, supporting the use of serious games for this group for home therapy and interactive games.

**Clinical Trials Registration:**

https://Clinicaltrials.gov, NCT04402034. Registered on May 20, 2020.

## Introduction

Individuals with Cerebral Palsy (CP) present motor disorders that are commonly associated with changes in sensation, learning, body perception, communication, behavior, and secondary complications such as epilepsy and musculoskeletal disorders that impair the individual’s functional performance (Bax et al., 2005). Considering these difficulties in different sensorimotor areas it is crucial for individuals with cerebral palsy to have continuous access to rehabilitation services (World Health Organization, 2004; Bax et al., 2005; Colver et al., 2014).

Although an effective rehabilitation program is important for people with cerebral palsy, in most countries, the resources available for adequate, regular rehabilitation for young people with neurological conditions are insufficient (World Health Organization, 2004). Moreover, with the new coronavirus (COVID-19), which has spread from human to human relentlessly and rapidly all over the world (Jakovljevic et al., 2020), access to health care services is even more limited, since most countries instigated a quarantine, i.e., through separation and restriction of movement of people who have potentially been exposed to a contagious disease to ascertain if they become unwell, the risk of infecting others is reduced (Brooks et al., 2020).

However, new technologies provide exciting opportunities for maintaining treatment for individuals with cerebral palsy through home-based telerehabilitation (HBTR) (Hosseiniravandi et al., 2020). A recent systematic review reveals evidence of HBTR to promote motor performance and self-care for children and adolescents with CP (Novak et al., 2020). HBTR offers the possibility of streamlining rehabilitation services, reducing therapist time, and permitting extended regular practice at times that are convenient for the users (Szturm et al., 2020). HBTR also provides the opportunity to increase the frequency of skills training, for people who find it difficult to regularly attend rehabilitation centers (Lloréns et al., 2015).

Serious-games, provide an interesting and effective way to support HBTR (da Silva et al., 2020a, Novak et al., 2020). Serious-games in non-immersive virtual reality (VR), can incorporate a range of learning elements with interactive motor and cognitive challenges, in an engaging environment providing opportunity for individuals with neurological disorders to participate in repetitive, adaptive, meaningful, and challenging motor skill practice (Gama et al., 2012; Levac et al., 2015; Lloréns et al., 2015; Schröder et al., 2019; de Moraes et al., 2020; Novak et al., 2020; Szturm et al., 2020). VR can motivate players to produce larger body movements and abandon a static position in front of the television or computer in order to play these interactive games (Crocetta et al., 2015). Gross motor function (Arnoni et al., 2019), motor performance (Leal et al., 2020), gait performance, balance abilities, leg strength (Cho et al., 2016) and reaction times (Pourazar et al., 2018) have been shown to improve when VR technology is used in children with CP. However, despite the potential benefits, evidence of the benefit of serious games (especially in HBTR) to date is limited, with studies tending to be of low methodological quality, across a range of methodologies, training doses and settings, making it difficult to draw uniform conclusions (Schröder et al., 2019).

Considering the above deliberation, this paper sets out to evaluate the feasibility and potential benefits of using computer software with a serious game implemented and evaluated completely remotely in participants with Cerebral Palsy in an HBTR during the quarantine period for COVID-19. Thus, we used a protocol with a coincident timing game to verify if participants with CP and a group of typically developing (TD) participants (TD group) (matched by age and sex) presented improved motor performance and increased physical activity intensity levels and motivation with a telerehabilitation program. We hypothesize that all participants would be able to present performance improvement (assessed through the game), with increases in physical activity intensity levels (assessed through rating of perceived exertion -RPE- scale) and motivation (assessed through visual analogue scale – (VAS) scale for satisfaction and motivation). However, considering all the sensorimotor difficulties that characterize CP, the improvements and benefits will be more evident for the TD group. If this hypothesis is confirmed, the results of this study will be relevant for the use of HBTR for individuals with CP.

## Materials and Methods

### Study Design

This paper was conducted during the strict quarantine period established by the state of São Paulo to reduce the transmission of COVID-19, between March 24 2020 and Jun 11 2020. Thus, this study used 100% telerehabilitation, in which the rehabilitation team and participants only had interaction through telephone contact, a communication application (WhatsApp), and software (MoveHero Software). The research was approved by Ethical Committee of the University of São Paulo, under CAAE: 03851012.7.0000.5390 and modified in March 2020 (adapted to COVID-19 research), registered on ClinicalTrials.gov NCT04402034, and reported in accordance with the Standard Protocol Items: Recommendations for Interventional Trials (SPIRIT) (Chan et al., 2013).

### Participants Recruitment

Researchers contacted 60 individuals with cerebral palsy through family members referred by the coordinators of two clinics in Brazil: Intertherapy and School-Clinic of the Fundação Hermínio Ometto (FHO-UNIARARAS), located in São Paulo state, and through posts on social media. The participants from TD group were recruited by social media (all from São Paulo state).

#### Inclusion Criteria

Participants were included if they had: age between 11 and 30 years old (adolescents and young adults); an agreement to participate in the research signed by themselves [by signing assessment form (Massetti et al., 2018)] and their legal guardians (by signing a consent form); a clinical diagnosis of CP carried out by a neuropediatric clinician (this information was provided by their parents); with GMFCS levels I to IV (these data were collected by video conference – the researcher contacted the family and was able to analyse motor function from a video).

#### Exclusion Criteria

Participants were excluded if they (1) did not understand the tasks − the understanding of the task was evaluated through 2 min of practicing the task (the individual was excluded if they did not understand the task during the first 2 min of practicing); (2) motor difficulties that impeded the completion of the virtual tasks (the individual was excluded if did not present motor ability to perform the task during 2 min); (3) did not have technology devices to perform the virtual task (computer or tablet) or to contact the researcher (cell phone or a second computer); and (4) were precluded from completing the task due to some technological failure (such as internet crash).

##### Contact and assessment scales

The parents of the individuals with CP were contacted by phone and were asked to help the participants to perform the task at home. The tasks were conducted by the researcher over the phone, via a video call. It is important to emphasize that all participants with CP and the Typically Developing (TD) group were assisted by their parents or their caregivers. First, they received a link with research information and the assent and consent form to fill out. The inclusion criteria were then checked by a researcher, and the Gross Motor Function Classification System (GMFCS) assessment was performed. After they agreed to participate, a link to access a questionnaire was sent with the Rating of Perceived Exertion (RPE) scale and sociodemographic information. When the parents and participants had completed the scales, the game platform was accessed, and the participant started the protocol. After finishing the intervention, the participants completed the visual analogue scale (VAS) scale for satisfaction and motivation (Heller et al., 2016;, Sung and Wu 2018).

### Materials and Apparatus

#### Instrument

In this study, we used a platform called MoveHero, available for free use in Portuguese https://movehero.com.br/ and English https://movehero.com.br/en/. The individual’s representant (family member) was required to access the Internet, and once they were online, create their own account, inserting the participant’s name and email and creating a password (all data collected were saved in the software system and only the principal investigator had access). The platform presents different levels of difficulty, so after the participant was connected to the platform, the researcher directed the participant to the protocol developed for this research.

**FIGURE 1:**
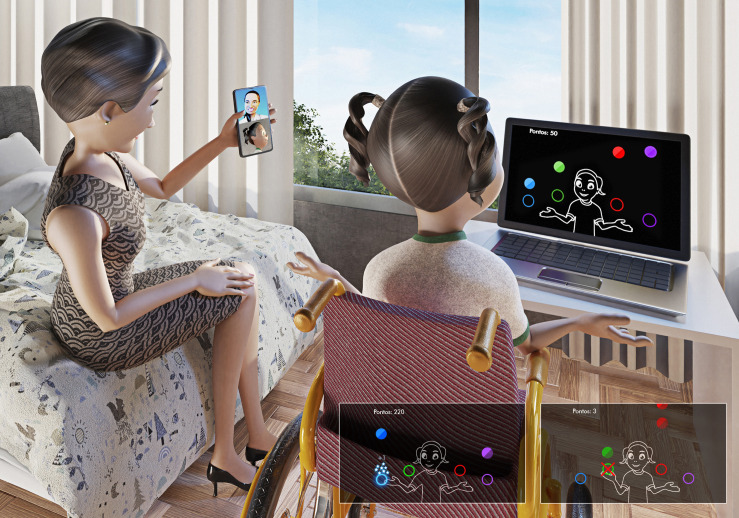
Participant positioning and game design.

Presented by Martins et al. (2019), MoveHero is considered a coincident timing task and presents several spheres falling down the computer screen, with a musical rhythm to increase engagement. The participant is positioned in front of a computer and when the game starts the webcam captures the participant’s movements and a representation of the player appears on the computer screen as an avatar (Figure 1). The goal of the game is to intercept all falling spheres using upper limb wave movements at the exact moment the spheres reach their specific target at the bottom of the computer screen. The game presents four columns with fixed parallel targets allocated at two height levels (e.g., two on the left - targets A and B; two on the right – targets C and D). The game also provides sensory feedback (visual - hit and miss feedback; auditory – anticipatory and delay error) – if the individual reaches the spheres correctly, the game presents feedback with the spheres changing the color of the target to blue, with little stars around it (hit information). On the other hand, if the participant does not reach the spheres correctly, the spheres change color to red and the letter X appears inside the target (miss information) together with a sound indicating an error.

#### Software Score

During the games, the participant can follow the score determined by the number of spheres hit (i.e., the information from each sphere hit appears in the bottom left side of the screen at all times) and at the end of each game the participant receives feedback with their total score (when the game is finished a total score appears in the middle of the computer screen).

#### Assessments

##### Characterization

(1) The Gross Motor Function Classification System (GMFCS): is a reliable and valid standard classification system of gross motor function for cerebral palsy that analyses an individual within 5 levels according to his/her ability to move, functional limitations, need to use assistive devices for walking, or need for a wheelchair (González-Alonso and Matía Cubillo, 2018).

(2) Sociodemographic Information: some questions are asked, regarding age, sex, income, level of CP, etc. (see Table 1) in order to understand the studied sample.

**TABLE 1:** It described the characterization of the group.

Variables		Group		*p*-value
		CP	TD	
		
		*Mean ± SD*	*Mean ± SD*	
**Weight**		48.3 ± 11.7	56.5 ± 21.8	**0.004**
**Height**		1.51 ± 0.14	1.60 ± 0.17	0.100
**Age**		17.8 ± 9.9	16.8 ± 8.4	0.532
		***n (%)***	***n (%)***	
**Sex**	Female	9 (41)	9 (41)	1.000
	Male	13 (59)	13 (59)	
**GMFCS**	I	6 (27)	–	–
	II	4 (18)	–	
	III	7 (32)	–	
	IV	3 (14)	–	
	V	2 (9)	–	
**Intellectual disability**	Yes	3 (13)	0 (0)	0.073
	No	19 (87)	22 (100)	
**Learning difficulty**	Yes	8 (36)	0 (0)§	**0.002**
	No	14 (64)	22 (100)§	
**Seizure**	Yes	4 (18)	0 (0)§	**0.036**
	No	18 (82)	22 (100)§	
**Sight disability**	Yes	11 (50)	0 (0)§	**<0.001**
	No	11 (50)	22 (100)§	
**Hearing disability**	Yes	2 (9)	0 (0)	0.148
	No	20 (91)	22 (100)	
**Therapy (type)**	Physiotherapy	14 (64)	0 (0)§	**<0.001**
	Occupational Therapy	1 (4)	0 (0)	
	Physical Activity	4 (18)	16 (73)§	
	None	3 (14)	6 (27)	0.385
**Hours of therapy**	1–2 h / week	8 (36)	3 (14)	
	3–5 h / week	8 (36)	10 (45)	
	6–9 h / week	1 (4)	2 (10)	
	>10 / week	2 (10)	1 (4)	
	none	3 (14)	6 (27)	
**During the COVID-19 pandemic, did your child stop any of the following therapy / activities?**	No	6 (28)	3 (14)	**0.013**
	Yes, totally	10 (45)	8 (38)	
	The activities were adapted by the therapists to be performed at home	5 (23)	1 (4)	
	I adapted the activities at home by myself	1 (4)	9 (43)§	
**Dominant hand**	Right	15 (68)	20 (91)	0.062
	Left	7 (32)	2 (9)	
**Does your child use a smartphone?**	Yes	15 (68)	17 (73)	0.401
	No	7 (32)	5 (27)	
**Does your child use a laptop?**	Yes	2 (9)	5 (27)	0.064
	No	20 (91)	17 (73)	
**Does your child use a tablet/iPad?**	Yes	4 (18)	3 (14)	0.680
	No	18 (82)	19 (86)	
**Does your child use a desktop?**	Yes	2 (9)	6 (27)	0.118
	No	20 (91)	16 (73)	
**Does your child use a videogame?**	Yes	3 (14)	6 (27)	0.262
	No	19 (86)	16 (73)	
**Does your child watch TV?**	Yes	5 (23)	2 (9)	0.216
	No	17 (77)	20 (91)	
**How long does your child use these devices (all together) per week?**	<2 h/week	1 (4)	1 (4)	0.747
	2–5 h/week	1 (4)	0 (0)	
	5–10 h/week	6 (28)	5 (23)	
	>10 h/week	14 (64)	16 (73)	
**Does your child use video-game?**	No	9 (41)	10 (46)	0.553
	Yes, 1–2 h/week	12 (55)	11 (50)	
	Yes, 2–3 h/week	0 (0)	1 (4)	
	Yes, >3 h/week	1 (4)	0 (0)	
**Does your child use Cell phone/tablet to play?**	Yes	16 (73)	16 (73)	1.000
	No	6 (27)	6 (27)	
**Use of Virtual Reality**	Yes	9 (41)	0 (0)§	**0.001**
	No	13 (59)	22 (100)§	

*CP, cerebral palsy group; TD, typical development group; SD, standard deviation; GMFCS: gross motor function classification scale. §*p* < 0.05 found by Bonferroni *post hoc* test in the chi-square comparisons.*

##### Outcome measures

(1) RPE scale: The Rating of Perceived Exertion was used to measure the subjective intensity of effort. The RPE is based on the sensations felt during exercise, such as muscle fatigue, increased heart rate, and increased breathing (Andrews et al., 2013). The RPE, although subjective, is used in several studies with the CP population (Maltais et al., 2004; Maanum et al., 2010; Runciman et al., 2016; Hjalmarsson et al., 2020) and can be considered a valuable indicator to monitor the tolerance to exercise and signalize imminent fatigue (ACSM).

(2) Motor performance: assessed during the game, through accuracy and precision of movement, as well as number of hits and mistakes.

(3) Motivation and satisfaction with the games and telerehabilitation were measured using a visual analogue scale (VAS) from 0 to 4.

### Intervention

#### Practicing the Task

Participants performed the task individually in their own homes with at least one family member helping and giving support throughout the protocol. The researcher contacted the family member using a video call and gave the following instructions (researcher interacted with the family member and the participant throughout the protocol): (1) place the computer on a table and login to the platform; (2) position the cell phone (to provide video call) on the side of the computer to receive instructions; (3) provide a comfortable sitting position in a chair (individual should be positioned at a distance of approximately 1.5 m meters from the computer monitor) and adjust the height according to the needs of the individual (if applicable, participants could use their own wheelchair); (4) after the participant was seated and connected to the platform, the researcher explained the task verbally to all participants and a 2 min demonstration of how to perform the game was given for the family member. Next, the family member was asked to setup the game for their child/adolescents to play; (5) after the demonstration, the family member was asked to pick up the mobile phone (to have the opportunity to move the phone and show the participant’s performance to the researcher during the protocol); and (6) thus, the therapist (by video call) instructed the participant to stay still and wait for the first sphere to appear on the screen. Once the first sphere appeared, the individual was required to move his or her hand in front of the camera to reach the sphere exactly at the moment coinciding with the target and the game continued with different spheres falling down the computer screen.

#### Intervention Game Protocol

**FIGURE 2:**
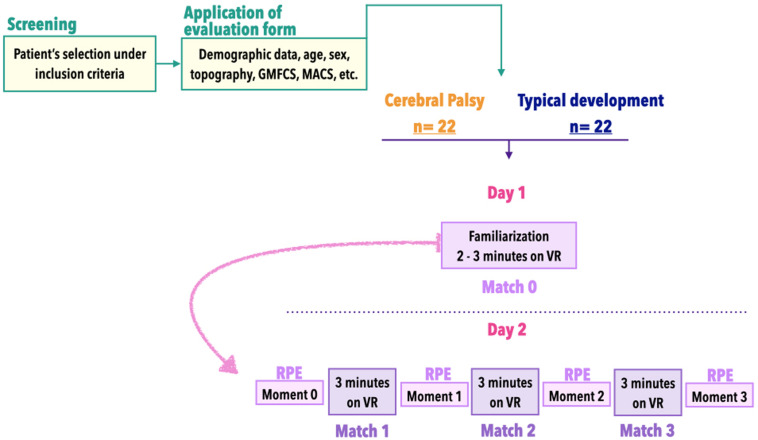
Overview of study design.

The MoveHero protocol was divided into 4 different matches (M0, M1, M2, and M3). M0 was the first contact with the task and was considered a familiarization phase to limit the interference caused by the use of new technologies. According to Lopez et al. (2016), the familiarization phase should be assessed *per se*, based on the principles of errorless learning. During familiarization the required actions are learned progressively in order to limit the production of errors and favor solid understanding of the rules of use (Lopez et al., 2016). Thus, in M0 all participants had the opportunity to learn how to play the game during a maximum of 3 min. After this familiarization period and on the second day, participants started M1, M2, and M3, each game consisting of 3 min of playing the MoveHero software with an interval of approximately 20 s between games to see the score and answer the RPE scale. The RPE scale was used at 4 moments: before starting M1 – baseline, after M1, after M2, and a final RPE after M3 (the study design depicted in Figure 2).

## Data Analysis

We considered the timing error provided by the game (in milliseconds) the dependent variable. The timing error was defined as the difference between the time the sphere started dropping and the time the individual managed to hit the target with the avatar’s hand. As used by de Mello Monteiro et al.(2014; 2017), Bezerra et al. (2018), and Martins et al. (2019), we analyzed the absolute error (AE), which demonstrates the accuracy of the movement; and the variable error (VE), which identifies the precision of the movement (for details about these errors, see de Mello Monteiro et al., 2017). As well as AE and VE we used as dependent variables the percentage of hits and misses and Rating of Perceived Exertion (RPE). As the assumption of normality was not met, non-parametric tests were used. We used Independent-Samples Mann-Whitney for comparisons between groups (CP and TD) and the Friedman test to compare matches (M0, M1, M2, and M3) and moments (Mo0, Mo1, Mo2, and Mo3), with Bonferroni as *post hoc* test. The effect size was calculated by using G^∗^Power software, version 3.1, and interpreted as *d* = 0.2 be considered a “small” effect size, 0.5 represents a “medium” effect size and 0.8 a “large” effect size (Lakens, 2013).

For the analysis of sample characterization, we used the independent samples *t*-test to compare groups (to attest homogeneity of groups) when the data were continuous and the chi-square test for categorical data. Values of *p* < 0.05 were considered significant. The statistical package used was SPSS (IBM Corporation, Armonk, NY, United States), version 20.0.

## Results

After being given an explanation of the task, 60 participants agreed to participate. Potential and interested volunteers underwent a detailed screening using the eligibility criteria and attended an initial selection for enrolment in the study. A total of 44 individuals were eligible and participated in this study, 22 of which had CP (14 males and 8 females, mean age = 19 years, ranging between 11 and 28 years.) and 22 typically developing individuals, matched by age and sex to the individuals with CP. Within the CP-group, there were 10 individuals with diparetic spasticity, 8 with right spastic hemiparesis, 8 with left spastic hemiparesis and 6 with choreoathetosis.

### Motor Performance – Absolute Error – AE

**FIGURE 3:**
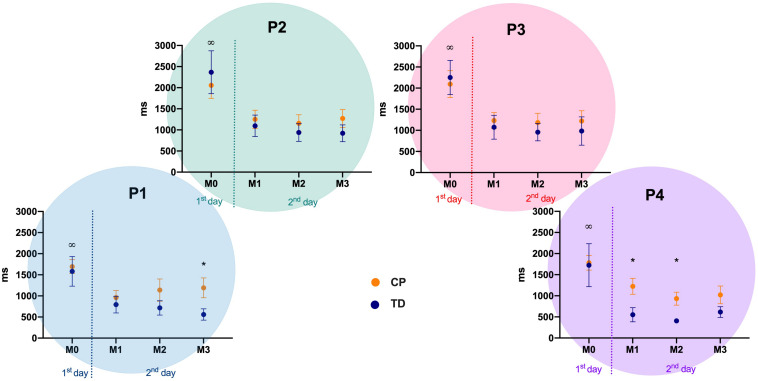
Absolute Error mean and standard error for both CP and TD-groups during familiarization phase (M0) and the three following matches (M1 to M3), in the four positions of the targets (P1 to P4). P1 to P4: positions of the targets of the MoveHero task; M0 to M3: Matches of the task, in which M0 is the familiarization phase and M1 to M3 are the three matches; CP, cerebral palsy group; TD, typical development group. *p < 0.05 between CP and TD groups; ^∞^*p* < 0.05 between match 0 and all other matches.

Differences were observed between CP and TD groups in position 1 (left) at match 3 (Mann-Whitney U: 131, *p* = 0.015, *d* = 0.68), and in position 4 (right) at match 1 (Mann-Whitney U: 77, *p* < 0.001, Cohen’s *d* = 0.84) and 2 (Mann-Whitney U: 118.5, *p* = 0.006, *d* = 0.78). The descriptive values are depicted in Figure 3.

Regarding comparisons within matches, the significant differences occurred within M0 and the other three matches in all positions (M0 versus M1: *P1* = 0.035, *d* = 0.84, *P2* = 0.006, *d* = 0.76, *P3* = 0.008, *d* = 0.82, *P4* = 0.002, *d* = 0.75; M0 versus M2: *P1* = 0.006, *d* = 0.73, *P2* = < 0.001, *d* = 0.87, *P3* = 0.002, *d* = 0.91, *P4* = < 0.001, *d* = 0.96; M0 versus M3: *P1* = 0.003, *d* = 0.82, *P2* = < 0.001, *d* = 0.83, *P3* = < 0.001, *d* = 0.79, *P4* = < 0.001, *d* = 0.82) (Figure 3).

### Motor Performance – Variable Error – VE

**FIGURE 4:**
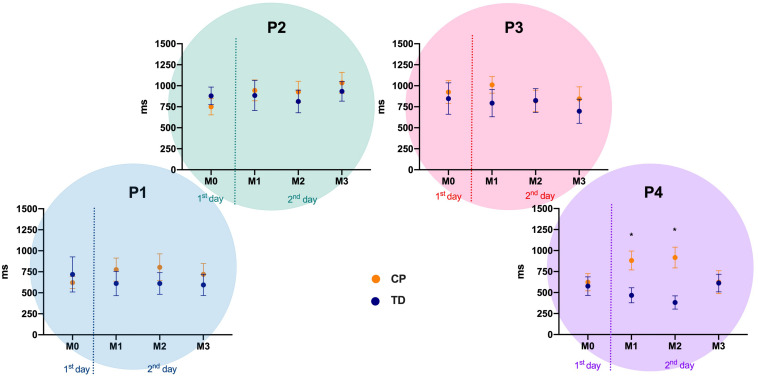
Variable Error mean and standard error for both CP and TD-groups during familiarization phase (M0) and the three following matches (M1 to M3), in the four positions of the targets (P1 to P4). P1 to P4: positions of the targets of the MoveHero task; M0 to M3: Matches of task, in which M0 is the familiarization phase and M1 to M3 are the three matches; CP, cerebral palsy group; TD, typical development group. *p < 0.05 between CP and TD groups.

There were differences between the CP and TD groups only in position 4 (right) at matches 1 (Mann-Whitney U: 118, *p* = 0.010, *d* = 0.70) and 2 (Mann-Whitney U: 102.5, *p* = 0.002, *d* = 0.89). The descriptive values are depicted in Figure 4.

Regarding comparisons within matches there were no significant differences between M0 and the other three matches in all positions for both CP and TD-groups (Figure 4).

### Motor Performance – Percentage of Hits

**FIGURE 5:**
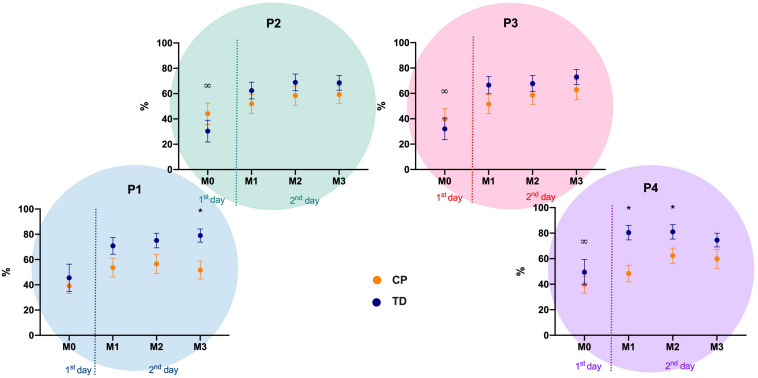
Mean and standard error of the percentage of hits for both CP and TD-groups during familiarization phase (M0) and the three following matches (M1 to M3), in the four positions of the targets (P1 to P4). P1 to P4: positions of the targets of the MoveHero task; M0 to M3: Matches of task, in which M0 is the familiarization phase and M1 to M3 are the three matches; CP, cerebral palsy group; TD, typical development group. *p < 0.05 between CP and TD groups; ^∞^*p* < 0.05 between match 0 and all other ma.

Similarly, to AE, there were differences between the CP and TD groups in position 1 (left) at match 3 (Mann-Whitney U: 346, *p* = 0.021, *d* = 0.73), and in position 4 (right) at matches 1 (Mann-Whitney U: 357, *p* = 0.001, *d* = 0.78) and 2 (Mann-Whitney U: 334, *p* = 0.002, *d* = 0.98). The descriptive values are depicted in Figure 5.

Regarding comparison within matches the significant differences occurred between M0 and some of the other three matches, depending of the position of the targets and position: (M0 versus M1: *P4* = 0.018, *d* = 0.64; M0 versus M2: *P2* = 0.003, *d* = 0.81, *P3* = 0.007, *d* = 0.85, *P4* = 0.001, *d* = 0.92; M0 versus M3: *P2* = 0.047, *d* = 0.84, *P3* = 0.001, *d* = 0.98, *P4* = 0.023, *d* = 0.75) (Figure 5).

### RPE, Game Score, Hits Percentage, and Misses Percentage (All Positions Together)

There were differences between groups for (a) RPE, (b) Score in the game, and (c) % of hits, at matches M1 [(a) Mann-Whitney U: 165, *p* = 0.051, *d* = 0.59; (b) Mann-Whitney U: 330.5, *p* = 0.038, *d* = 0.64; (c) Mann-Whitney U: 346, *p* = 0.015, *d* = 0.72], M2 [(a) Mann-Whitney U: 132, *p* = 0.009, *d* = 0.80; (b) Mann-Whitney U: 332, *p* = 0.014, *d* = 0.71; (c) Mann-Whitney U: 330, *p* = 0.039, 0.60], and M3 [(a) Mann-Whitney U: 133.5, *p* = 0.004, 0.84; (b) Mann-Whitney U: 383.5, *p* = 0.001, *d* = 0.92; (c) Mann-Whitney U: 316, *p* = 0.061. *d* = 0.52]. In % of misses there was a significant difference only at M3 (*p* = 0.040, *d* = 0.68).

**FIGURE 6:**
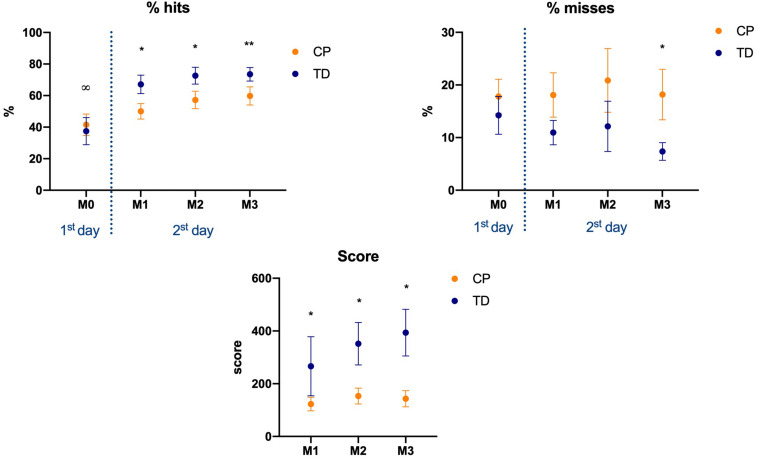
Mean and standard error of percentage of hits, misses, and the continuous score, for both CP and TD-groups during the familiarization phase (M0) and the three following matches (M1 to M3), in the four positions of the targets (P1 to P4). M0 to M3: Matches of task, in which M0 is the familiarization phase and M1 to M3 are the three matches; CP, cerebral palsy group; TD, typical development group. *p < 0.05 between CP and TD groups; ^∞^*p* < 0.05 between match 0 and all other matches.

For % of hits there were significant findings within M0 and all the other three matches (M0 versus M1, *p* = 0.011, 0.73; M0 versus M2, *p* < 0.001, 0.96; M0 versus M3, *p* < 0.001, 0.97). For matches score there were significant effects between M1 and M2 (*p* = 0.001, *d* = 0.82) and M1 and M3 (*p* < 0.001, *d* = 0.90). For % of misses no significant effects were found (Figure 6).

**FIGURE 7:**
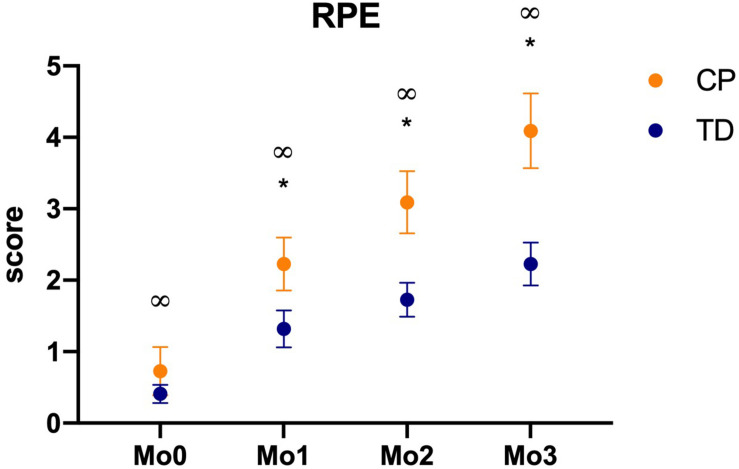
Mean and standard error of RPE score, for both CP and TD-groups before first match (Mo0), after first (Mo1), second (Mo2), and third matches (Mo3), in the four positions of the targets (P1 to P4). M0 to M3: Moments of task, in which M0 is the familiarization phase and M1 to M3 are the three games; CP, cerebral palsy group; TD, typical development group. *p < 0.05 between CP and TD groups; ^∞^*p* < 0.05 between moments.

A very interesting result was found regarding RPE (Figure 7). When comparing moments within them, there are significant effects between all moments for both CP and TD-groups (Mo0 versus Mo1, *p* = 0.001, *d* = 0.87; Mo1 versus Mo2, *p* < 0.001, 0.93; Mo2 versus Mo3, *p* < 0.001, *d* = 0.95) (Figure 7).

### Game Engagement

**FIGURE 8:**
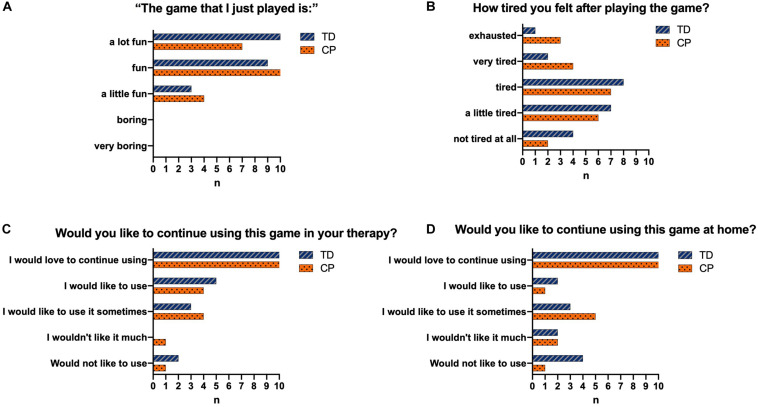
Representation of the answers of the individuals from both CP and TD groups regarding their satisfaction with the game. TD, Typical Development; CP,Cerebral Palsy; *n* = number of participants who chose this option. **(A)** shows the results of the participants who found the game fun. **(B)** shows the results of the participants who felt tired after the intervention. **(C)** shows the results of the participants who would like to continue using the game in the rehabilitation clinic. **(D)** shows the results of the participants who would like to keep using the game at home.

Regarding engagement, Figure 8 shows the results of the participants who found the game fun (Figure 8A), felt tired after the intervention (Figure 8B), would like to continue using the game in the rehabilitation clinic (Figure 8C), and would like to keep using the game at home (Figure 8D).

### Correlation

In order to establish the influence of age, body mass index, level of CP and time spent in technological devices over the increase in RPE (delta: Mo3-Mo0) and performance in the game, we conducted a correlation analysis. It showed no influence of any of the independent variables over the dependent variables.

## Discussion

Due to the world problem with quarantine and the impossibility of individuals with CP maintaining traditional rehabilitation activities (i.e., presential rehabilitation), the current study investigated the possibility of setting up and delivering a home-based telerehabilitation (HBTR) protocol using a serious game platform to improve motor performance, and increase physical activity and motivation in individuals with CP during a strict quarantine period. Our initial hypothesis was partially supported as all participants were able to engage with the therapy remotely and demonstrated improved performance, increasing their physical activity in this cross-sectional study, providing a positive base for developing HBTR in this group. However, contrary to our hypothesis the TD group did not present better motor performance in all aspects of the protocol compared to the CP group opening opportunities for motivating competitions with TD peers and siblings. These results will be explained below.

### Motor Performance

Considering motor performance, all participants presented improvement when comparing M0 (familiarization match) with other matches (M1, M2, and M3). According to Wadden et al. (2019), a higher degree of challenge, when the motor response or task-demands are more complex, affects the conditions of learning. Therefore, to provide improvement in performance, individuals first need to know how to perform the task. Thus, as we used an unknown task, in our study we provided a familiarization match, in order to allow participants to understand the task. Our results showed improvement from the familiarization match (M0) to the first practice match (M1), which means that both groups understood the task and were able to play the MoveHero game, although they did not improve performance during the practice. This supports the use of a familiarization practice session for CP and TD group.

The performance improvement was not observed continuously, i.e., there was no improvement in performance considering absolute error and number of hits between M1, M2, and M3 matches. It is well known that after familiarization, to execute a task and acquire fluency of movement, the participant requires practice with repeated experience to achieve automation, accuracy, and precision of movement (Kawahira et al., 2004; Gabitov et al., 2014; Magallón et al., 2016). Thus, we can speculate that a longer period of practice (more days practicing), and greater experience with the task would be positive to learn how to use feedback from the virtual environment (with no haptic feedback) and could provide higher automation to the task, with performance improvement, and, actually that is what happens in serious game players; the more they play, the better they get.

Considering performance comparisons between groups, the TD group performed better than the experimental group only in some positions and matches, contrary to our initial hypothesis, as we expected better performance from the TD group in all positions and matches. Our hypothesis was based on the studies of de Mello Monteiro et al. (2014), Martins et al. (2019), and Prado et al. (2017) that compared motor learning between individuals with CP and their typically developing peers during tasks in a non-immersive VR environment and identified that timing accuracy was significantly worse in individuals with CP than in the TD group.

However, in our study, the TD group presented better performance only in lateral targets considering absolute error and target hits (i.e., the TD group presented better performance in M3 in A1 position – left side; and M1 and M2 in A4 position – right side). As presented before, in all other matches and positions we did not find statistical differences. This is quite intriguing and it is interesting that all the difficulties that characterize individuals with CP caused differences from the TD group only in the lateral targets and in some matches. This data provides support for the use of serious games in young people with CP and the possibility for direct competition with TD young people on some serious games activities. It is probable that the CP difficulties such as permanent neurological impairment (Panteliadis et al., 2015) associated with significant sensorimotor dysfunction (Reid et al., 2015), muscular weaknesses (Soares et al., 2019), high levels of co-activation (Soares et al., 2019), abnormal muscle recruitment with spasticity (Booth et al., 2019; Ko et al., 2020) and slowness of movement (da Silva et al., 2020b) disturbed the performance of individuals with CP when they were required to use a bigger range of movement with accuracy (i.e., targets more distant in the lateral position needed bigger range of movement and accuracy). According to Fernani et al. (2017) who evaluated CP and a TD group in a computer task to verify movement speed-accuracy trade-off, by changing width and distance of targets, the authors identified a worse movement time in the CP group when compared with typically developing individuals and the authors suggested that the difference could be explained by considering specific motor control difficulty, which means that individuals with CP presented worse accuracy and velocity of movement with more distant objects than their TD peers, which could explain the difficulty with the lateral targets.

### Physical Activity

Another important finding was that the serious game led to increasing rating of perceived exertion, measured through the Borg scale, suggesting that the game used promotes an increase in physical activity. A review by Mitchell et al. (2012) argued that to date the emphasis has been on establishing the feasibility of serious game as a therapeutic modality for rehabilitation and that there is less research investigating the usability of serious game systems to increase physical activity in people with disabilities. This is a very important finding due to the growing evidence of the higher prevalence of metabolic syndrome, cardiovascular disease risk factors, and autonomic nervous system dysfunctions in adults with CP, because of their limited mobility since childhood, which leads to sedentary behavior, disposing them to chronic disorders such as heart conditions and hypertension (Heyn et al., 2019; Katz-Leurer and Amichai, 2019; da Silva et al., 2020a). This emphasizes the importance of new research with a view to the practice of physical activity in individuals with CP, since more active behaviors contribute to health promotion.

#### Differences Between Moments of Physical Activity Practice

Considering differences between moments, we believe that this increase in the rate of perceived exertion in both groups could be due to high engagement promoted by serious game tasks. For this engagement to have occurred, three elements are essential: Repetition, Feedback, and Motivation (Pereira et al., 2014), as explained below:

Repetition: Considering motor learning and level of physical activity, one of the essential factors for success is repetition, and serious games have the potential to increase the repetition of the task, providing different intensities of movement and functional activities (French et al., 2016). Thus, as the participants execute body movements, such as the repetition of muscle contractions (Alvarez et al., 2017) during the serious game, the level of physical activity will gradually enhance (Gomes et al., 2019) with an adaptation to an aerobic activity and improvement in physical capacity and efficiency of the cardiorespiratory system (Powell et al., 2011). This is in accordance with Sheehy et al. (2016), who stated that serious game is enjoyable and may motivate patients to perform more repetitions of their movement in a ludic environment, with goal-oriented movements, contributing to increased intensity and, consequently, physical activity.

Feedback: Our study did not use tactile or haptic feedback, since these experiences in VR require gloves or other expensive devices that were not available and the main objective of the MoveHero software used, is to provide a possibility for promotion of physical activity and/or rehabilitation with the lowest possible financial cost, by using auditory and visual feedback from just a computer or tablet and internet connection. The software provided constant visual and auditory feedback for hit or miss and, although this was not enough to improve performance during our protocol, it was sufficient to create an engaging environment, maintaining the motivation and participation of the participants (Shafer et al., 2019).

Motivation: the music and coloured spheres falling down are additional motivation factors and the most important contribution to guarantee player engagement in order to increase the rate of perceived exertion. According to Villiger et al. (2013), sensorimotor network activation can be enhanced when the individual plays interactively and with high motivation. Fortier et al. (2011) guarantee that motivation is an important factor to engage in physical activity interventions.

In this context, our results (Figure 8) demonstrate that all participants liked the game and the majority reported that they would like to keep practicing at home as a rehabilitation possibility (for the CP group) or physical activity intervention (for TD group). It is probable the MoveHero software provides the player with a new experience and a way of having fun at home. In a study by Bryanton et al. (2006) the authors observed that children are often not compliant in following a conventional home exercise program because they find the exercise meaningless and uninteresting. Therefore, the VR task allows the participant to experience stimulating environments, providing challenging tasks (Booth et al., 2019), fun, interest in making their scores higher (Zangirolami-Raimundo et al., 2019) and autonomy to the player, optimizing their motivation to continue practicing and allowing them to feel “good” about themselves (Wulf and Lewthwaite, 2016), with maintenance of movement practice of the task and, consequently, improved physical activity.

#### Differences Between Cerebral Palsy and TD Group in Physical Activity Level

Another interesting result was that the CP group presented a higher rate of perceived exertion than the TD group (Figure 8). The first justification for this result is that, considering no difference between groups in motor performance in most positions, as presented before, the individuals with CP expended more energy to finish tasks requiring body movements than typically developing individuals. According to Kerr et al. (2008) individuals with CP have been known to require greater energy consumption in some tasks as a consequence of the spectrum of the condition, such as spasticity and impaired postural control. It is probable the difficulty in practicing a sequential motor task in individuals with CP (Steenbergen et al., 2013) and the use of a strategy to avoid muscular perturbation caused by movement execution while reaching a target (Soares et al., 2019) were compensated by a higher neuromuscular cost (Fernani et al., 2017). Thus, during a new task, individuals with CP tend to avoid a bad performance and, consequently, need to pay more attention to motor tasks that are not automatic, (Krajenbrink et al., 2018) which probably increases the rate of perceived exertion.

A second justification is that individuals with CP participate in leisure-time physical activities less often, with less intensity, and with reduced diversity than their typically developing peers (Reedman et al., 2017). A review by Carlon et al. (2013) showed that young people with cerebral palsy performed significantly less habitual physical activity with consequent lower physical conditioning, and poor level of cardiorespiratory fitness when compared to a peer TD group.

### Conclusions and Clinical Considerations

Considering the importance of being active and maintaining an exercise routine as an essential target for physical health even during quarantine (Lippi et al., 2020), new possibilities to be active inside the home are essential, especially for people with CP in order to avoid cardiovascular disease risk factors, metabolic syndrome, and autonomic nervous system dysfunctions. In this context, our results show that active video games can elicit increased energy expenditure and translate into increased physical activity, representing a powerful tool to be used by therapists and families, such as home-based telerehabilitation.

We believe that another great differential of our study is that the use of a serious game in a non-immersive virtual reality software provides the possibility of a home-based telerehabilitation that can include not only the participation of the health professional, but also of their family, which is preconized for a successful intervention. Adult supervision of motor skills is required for ongoing motivational purposes and to support the cognitive and problem-solving processes during practice (White, 2017), encouraging the individuals with CP to participate and improve. Therefore, the possibility for parents and therapists to work together (and support each other) to adjust the difficulty of the task, and provide security during the practice was essential for understanding the task and increased physical activity. According to Cramer et al. (2019) and Gibbs and Toth-Cohen (2011), although more studies are needed on home-based telerehabilitation provided by a rehabilitation team working together with parents and patients, it seems to be safe, is rated favourably by patients, is associated with excellent treatment adherence, and produces substantial gains in function as well as interventions delivered in clinics.

### Limitations and Future Studies

Although we found interesting results, we can point out some limitations of the present study: (1) this was a 1 day protocol and a protocol with training lasting at least 10 days could provide better results in improving performance, physical activity, and adherence to serious game as Home-based telerehabilitation; (2) we did not analyze patterns of movement, because although these data could be important to identify quality of movement, they are difficult to assess in a telerehabilitation intervention; (3) we used a classification system to identify level of motor difficulty in the CP group (GMFCS), but it would be interesting to develop an assessment to be used for telerehabilitation, as this was proven to have potential for future interventions; (4) we should have measured the therapist and family influence during the practice, as these data could be important to define future interventions; and (5) this study misses a control group that would perform conventional interventions in telerehabilitation (without serious games).

## Data Availability Statement

The raw data supporting the conclusions of this article will be made available by the authors, without undue reservation.

## Ethics Statement

The studies involving human participants were reviewed and approved by the Ethics Committee of the University of São Paulo, under CAAE: 03851012.7.0000.5390. Written informed consent to participate in this study was provided by the participants’ legal guardian/next of kin.

## Author Contributions

TS designed the study, performed the statistical analyses, interpreted the data, and revised the manuscript critically for intellectual content. PS, EV, AS, MC, and AF collected patient data and drafted the manuscript. AS and CA drafted the manuscript. ED provided assistance on patient data collection and revised the manuscript. LA and MB developed the game used and revised the manuscript. HD revised the manuscript critically for intellectual content. CM coordinated the study, drafted the manuscript, and revised the manuscript critically for intellectual content. All authors read and approved the final manuscript.

## Conflict of Interest

The authors declare that the research was conducted in the absence of any commercial or financial relationships that could be construed as a potential conflict of interest.

## Abbreviations

AE: absolute error
CP: cerebral palsy
GMFCS: gross motor function classification system
HBTR: home-based telerehabilitation
RPE: rating perceived exertion
TD: typical development
VAS: visual analogue scale
VE: variable error
VR: virtual reality.
